# Abnormal expression of histone acetylases in CD8+ T cells of patients with severe aplastic anemia

**DOI:** 10.1002/jcla.24339

**Published:** 2022-03-10

**Authors:** Weiwei Qi, Yu Zhang, Yachen Wang, Huaquan Wang, Rong Fu, Zonghong Shao

**Affiliations:** ^1^ Department of Hematology Tianjin Medical University General Hospital Tianjin China

**Keywords:** CD8+T cells, HATs, HDACs, IFN‐γ, severe aplastic anemia

## Abstract

**Introduction:**

We aimed to investigate the balance between the mRNA levels of histone acetyltransferases (HATs) and histone deacetylases (HDACs) in CD8+ T cells of patients with severe aplastic anemia (SAA).

**Methods:**

Twenty untreated SAA patients, 18 remission SAA patients (R‐SAA), and 22 normal controls were evaluated. The mRNA expression levels of HATs, HDACs, and *IFNG* in CD8+ T cells were measured by real‐time quantitative reverse transcription polymerase chain reaction.

**Results:**

Histone acetylase *EP300* and *CREBBP* mRNA levels were significantly elevated in CD8+ T cells of SAA patients compared with the normal controls (both *p* < 0.05). No significant differences were observed in *HDAC1* and *HDAC7* mRNA between SAA patients and the normal controls. There was an obvious positive correlation between *IFNG* and *EP300* (*r* = 0.5126, *p* < 0.01), and *CREBBP* (*r* = 0.4663, *p* < 0.05), respectively, in SAA and R‐SAA patients. In addition, *EP300* and *CREBBP* mRNA levels were clearly correlated with clinical parameters of peripheral blood and bone marrow in those patients.

**Conclusion:**

Our findings suggest that *EP300* and *CREBBP* are increased in CD8+ T cells of SAA patients and are correlated with disease severity. The imbalances in HATs and HDACs may play a role in activating CD8+ T cells to promote the immune pathogenesis of SAA.

## Graphical abstract

Increased expression of histone acetylation enzymes *EP300* and *CREBBP* led to increased histone acetylation levels in CD8+ T cells of severe aplastic anemia (SAA) patients, which was correlated with abnormal activation of CD8+ T cells and the severity of SAA.

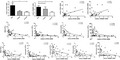

## 1 INTRODUCTION

Severe aplastic anemia (SAA) involves the immune‐mediated destruction of hematopoietic stem cells and is characterized by pancytopenia and bone marrow failure. The pathogenesis of SAA is closely related to the damage of hematopoietic cells by increased CD8+ T lymphocytes.^1^ Moreover, previous studies have demonstrated that Th1/Th2 imbalance (enhanced Th1) and the excessive secretion of type I lymphokines (IL‐2, IFN‐γ) may cause the increased number and hyperfunction of CD8+ T cells.^2^ Therefore, in this study, we focused our investigation on the relationship between histone acetyltransferases (HATs) and histone deacetylases (HDACs) in CD8+ T cells of SAA.

Epigenetic refers to the heritable alterations that affect gene transcriptional activity and gene expression without alterations in DNA sequences. Studies have shown that destruction of gene expression patterns that are controlled by epigenetics can lead to autoimmune diseases, cancers, and various other diseases.^3^ According to the different mechanisms of action, epigenetics can be divided into four aspects: DNA methylation, covalent modification of histones, chromosome remodeling, and microRNA expression. Covalent modifications of histones include methylation, acetylation, phosphorylation, ubiquitination, and glycosylation. Histone acetylation occurs at the relatively conserved lysine site at the amino terminus of histones H3 and H4 and is involved in many biological processes such as gene transcriptional regulation, cellular function regulation, inflammation and metabolism, and immune response. Histone acetylation is a reversible epigenetic modification that is regulated by HATs and HDACs. HATs can activate gene transcription by catalyzing histone acetylation and resulting in the loosening of chromatin structure; HDACs cause silencing of gene transcription by chromatin condensation due to histone deacetylation.^4^

Our previous study^5^ revealed that the amount of histone H3 acetylation was significantly increased in the CD8+ T cells of untreated SAA patients. In addition, the amount of histone H3 acetylation in CD8+ T cells was significantly and negatively correlated with absolute neutrophil count (ANC), proportion of reticulocytes, ratio of CD4+ to CD8+ T cells in peripheral blood, and percentage of erythroid cells in bone marrow. The abnormal acetylation of histone H3 in CD8+ T cells may be one of the reasons for the overactivation of CD8+ T cells.

Based on previous studies, we investigated the cause of abnormal histone H3 acetylation. In this study, we detected the mRNA expression levels of HATs and HDACs in CD8+ T cells of SAA patients and their relationship with the severity of the disease. The objective was to elucidate the relationship between histone acetylation of CD8+ T cells and immune pathogenesis in SAA patients.

## 2 MATERIALS AND METHODS

### 2.1 Patients

A total of 20 newly diagnosed SAA patients (median age 40.5, range 15–79 years old) who were previously untreated and 18 recovering SAA (R‐SAA) patients (median age 30, range 11–64 years old) were enrolled between the period of October 2017 and September 2019. The patients were hospitalized at the Hematology Department of Tianjin Medical University General Hospital (Tianjin, China). The diagnosis was performed according to the 2009 International AA Study Group criteria^6^: marrow cellularity <25% (or 25%–50% with <30% residual hematopoietic cells), and two of three parameters of neutrophil count <0.5 × 10^9^/L, platelet count <20 × 10^9^/L, and reticulocyte count <20 × 10^9^/L. Both abnormal chromosomes and PNH clones were absent from all patients. Patients were excluded who were diagnosed with congenital marrow failure or company with other autoimmune diseases. R‐SAA required transfusion independence after treatment with immunosuppressive therapy that included anti‐human thymocyte immunoglobulin (ATG) and cyclosporin A. A total of 22 normal controls were also enrolled, including 15 males and 7 females (median age 40.5, range 24–77 years old). Routine blood examinations were normal in all controls, and potential participants were excluded whether they had autoimmune diseases, cancers, or infections. This study was approved by the Ethics Committee of Tianjin Medical University. Written informed consent was provided by all patients and normal control in accordance with the Helsinki Declaration.

### 2.2 Sorting of CD8+ T lymphocytes

Ten milliliters of peripheral blood samples collected in EDTA anticoagulant tubes from each subject were used for mononuclear cell isolation by density gradient centrifugation with Ficoll solution (Solarbio) according to the manufacturer's instructions. Twenty‐microliter human CD8 MicroBeads (Miltenyi Biotec) were then added per 10^7^ total cells. The samples were incubated at 4°C for 15 min and washed with PBS, and the cells in suspension were passed through an MS column in a magnetic field, then detached the column from the magnet, added 1 ml wash buffer, and forced the plunger (in the same package as the column) to make the buffer through the column rapidly. Purity of CD8+ T cells was measured by CytoFLEX flow cytometry (Beckman Coulter) with anti‐CD8‐FITC antibody (BD Biosciences). The residual cells were centrifuged at 400× g for 5 min and used for the subsequent experiments.

### 2.3 RNA isolation, cDNA synthesis, and real‐time quantitative reverse transcriptase PCR (RT‐PCR)

Total RNA was extracted from the purified CD8+T cells using TRIzol reagent (Invitrogen Life Technologies). The quality and purity of the isolated total RNA was detected using a NanoDrop ND‐1000 (Thermo Fisher Scientific) at 260 and 280 nm. RNA with a 260/280 ratio ranging from 1.8 to 2.0 was accepted for the subsequent reverse reaction. The extracted RNA was reverse‐transcribed using a reverse transcription kit (TaKaRa) according to the manufacturer's instructions. The reverse transcription reaction was performed at 37°C for 15 min and 85°C for 5 s. The relative mRNA expression levels of HAT (*EP300* and *CREBBP*), HDAC (*HDAC1* and *HDAC7*), and interferon‐gamma (*IFNG)* were detected by RT‐PCR. *ACTB* was used as the housekeeping gene. The primers used for this analysis are listed in Table 1. Reactions were performed using the Bio‐Rad iQ5 Real‐Time System and SYBR Premix Ex Taq (TaKaRa). The relative expression levels of target genes were calculated using the 2^−ΔΔCt^ method.

**TABLE 1:** Primer sequences used for RT‐PCR

Gene	Forward (5′−3′)	Reverse (5′−3′)
P300	GCAGCATCACATGCAACAGA	AGGAGTCGCTGCTGATAGGC
CBP	AGCAGCCAGCATTGATAACAGAGTC	AATCCTCCTCCATCATCTCAGACCTG
HDAC1	GCCAATGCTGAGGAGATGACCAAG	GCCACAGAACCACCAGTAGACAAC
HDAC7	TGCACCACCACCTCTTCCTAGC	ACTTCGCTTGCTCTTGTCCTTGTG
IFN‐γ	AGTGATGGCTGAACTGTCGC	ACTGGGATGCTCTTCGACCT
β‐actin	CATGTACGTTGCTATCCAGGC	CTCCTTAATGTCACGCACGAT

### 2.4 Collection of clinical data

The complete blood counts of the SAA and R‐SAA patients were collected, including white blood cells (WBCs), hemoglobin (Hb) level, platelets (PLT), proportion of neutrophils and lymphocytes, ANC, absolute lymphocyte count (ALC), proportion of reticulocytes, and absolute reticulocyte count (ARC). In addition, the percentage of bone marrow granulocytes and lymphoid and erythroid cells, and the number of megakaryocytes in SAA and R‐SAA patients were collected.

### 2.5 Statistical analysis

SPSS 20.0 software was used for statistical analyses. Quantitative data are showed as mean ± SEM (mean ± standard error of mean). Significance of differences in quantitative parameters between groups were determined using the Student's *t*‐test or nonparametric test (Kruskal–Wallis). The Spearman coefficient correlation was used to analyze correlations among various parameters. *p* < 0.05 was considered statistically significant.

## 3 RESULTS

### 3.1 Purity of CD8+ T cells

**FIGURE 1:**
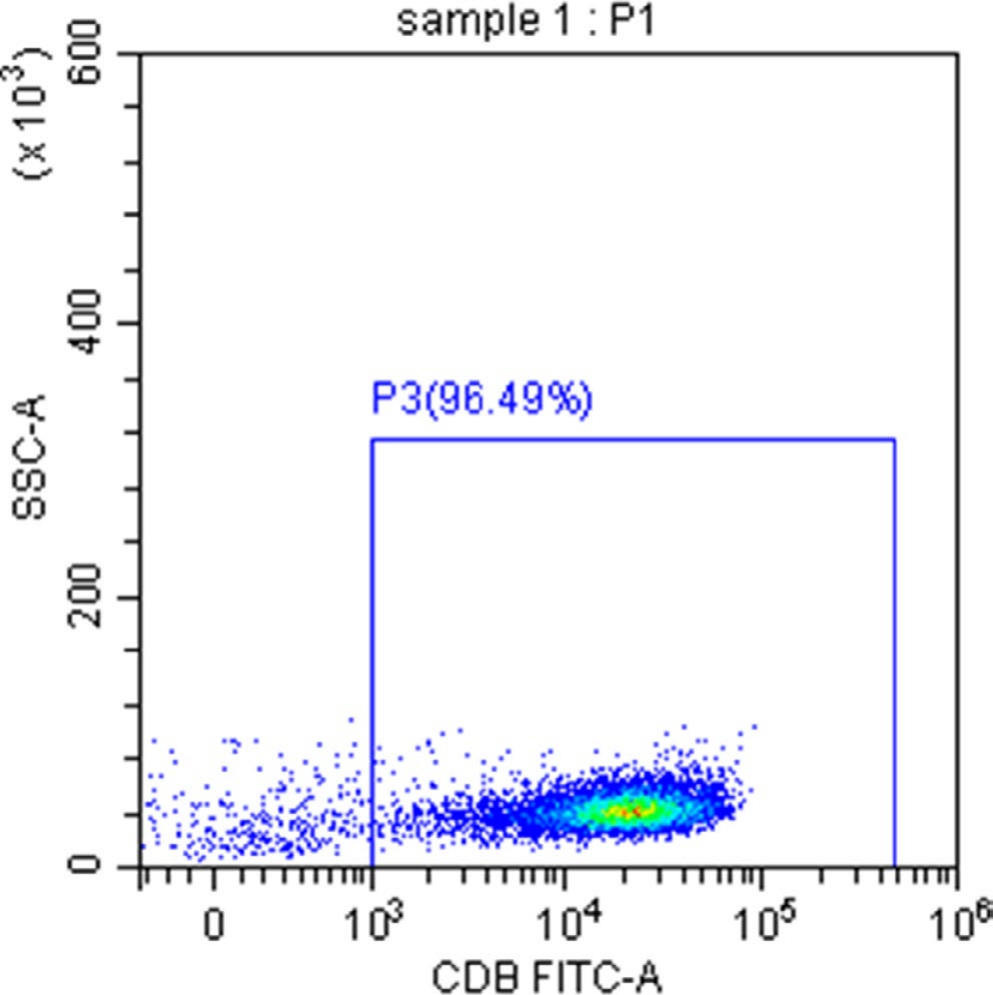
The purity of CD8+T cells isolated from peripheral blood mononuclear cells was more than 90%

The purity of sorted CD8+ T cells isolated from the peripheral blood of SAA patients, and the normal controls were more than 90% measured by flow cytometry (Figure 1).

### 3.2 *EP300* and *CREBBP* expression were increased in CD8+ T cells of SAA patients

**FIGURE 2:**
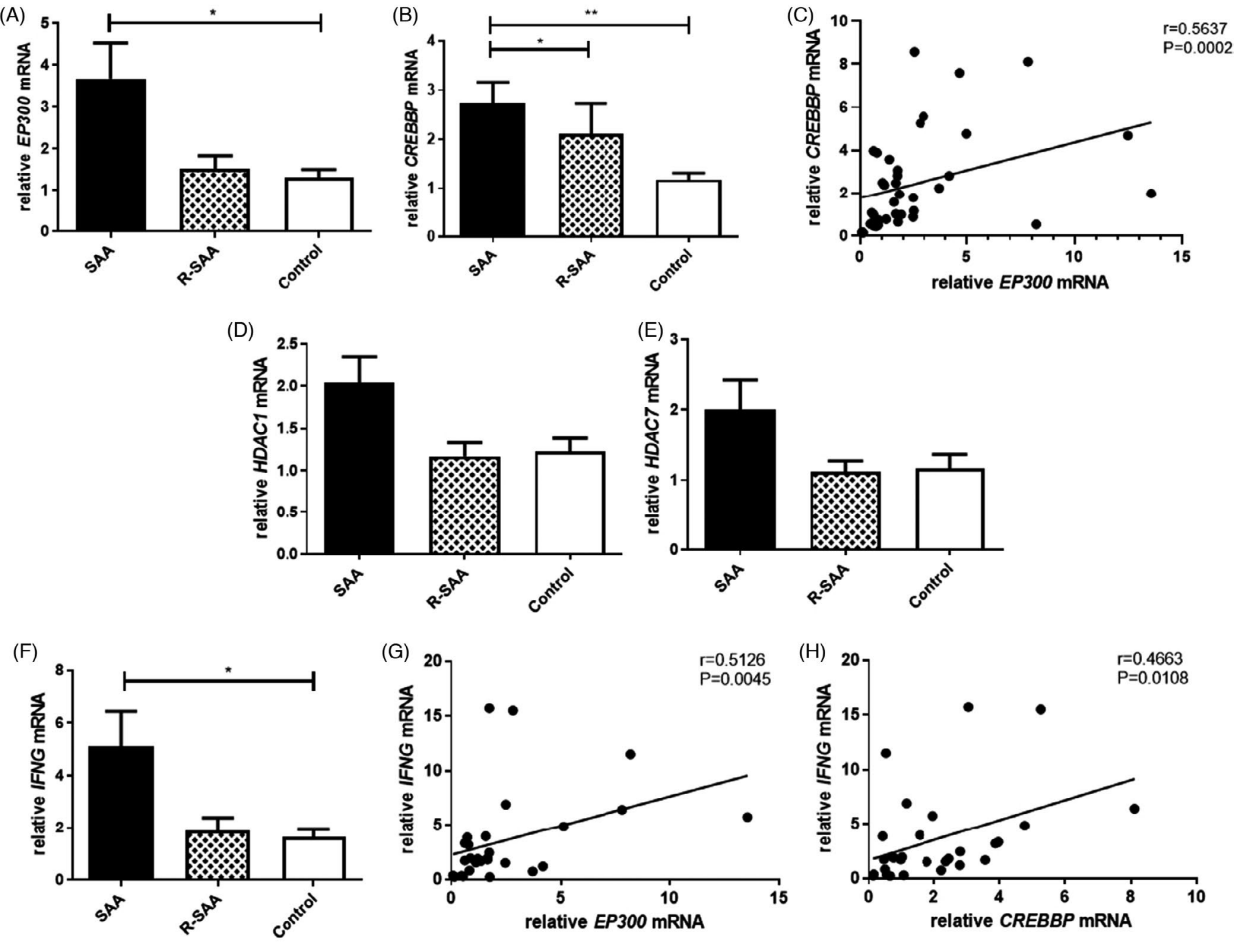
Relative mRNA levels of HATs, HDACs, and *IFNG* in CD8+ T cells between the three groups. HATs—histone acetyltransferases; HDACs—histone deacetylases

The mRNA expression level of *EP300* was significantly increased in CD8+ T cells of SAA patients compared with the controls (3.667 ± 0.862 vs. 1.300 ± 0.202; *p* < 0.05), while it was also higher than R‐SAA with no statistical significance (3.667 ± 0.862 vs. 1.524 ± 0.304; *p* > 0.05), and there was no significant difference between R‐SAA patients and the controls (Figure 2A). For *CREBBP*, the mRNA levels of untreated SAA patients, R‐SAA patients, and the normal controls were 2.742 ± 0.422, 2.119 ± 0.615, and 1.178 ± 0.137, respectively. The relative *CREBBP* mRNA levels of untreated SAA patients were significantly higher than those of R‐SAA patients and the controls (both *p* < 0.05), while the difference was not statistically significant between R‐SAA patients and the controls (Figure 2B). Interestingly, we discovered that relative *EP300* mRNA expression was positively and significantly correlated with *CREBBP* mRNA expression in CD8+ T cells of SAA and R‐SAA patients (*r* = 0.5637, *p* = 0.0002; Figure 2C).

### 3.3 No significant difference was observed in HDACs in CD8+ T cells between SAA patients and controls

When examining the mRNA expression of HDACs, we found that *HDAC1* mRNA was increased in SAA patients compared with R‐SAA patients and the normal controls (2.041 ± 0.305 vs. 1.167 ± 0.163 vs. 1.225 ± 0.162), but without significant differences (Figure 2D). Furthermore, the relative *HDAC7* mRNA levels of untreated SAA patients were higher than that of R‐SAA patients and the controls (1.998 ± 0.426 vs. 1.123 ± 0.152 vs 1.161 ± 0.204), but the differences were also not significant (Figure 2E).

### 3.4 Correlation between HATs and IFN‐γ in CD8+T cells of SAA patients

As shown in Figure 2F, the mRNA expression of *IFNG* in the CD8+ T cells of untreated SAA patients was significantly increased compared to that of the normal controls (5.124 ± 1.320 vs. 1.622 ± 0.319; *p* < 0.05), while it was also increased in R‐SAA patients but without a significant difference (1.890 ± 0.495 vs. 1.622 ± 0.319; *p* > 0.05). We then investigated the correlation between HATs and *IFNG* in CD8+ T cells of SAA patients. The results showed that there was a significant positive correlation between *IFNG* and *EP300* mRNA expression levels in CD8+ T cells of SAA and R‐SAA patients (*r* = 0.5126, *p* = 0.0045; Figure 2G). Similarly, *IFNG* mRNA expression was also positively correlated with *CREBBP* mRNA expression (*r* = 0.4663, *p* = 0.0108; Figure 2H).

### 3.5 Correlation between HATs and clinical parameters in SAA patients

The basic information and clinical parameters of SAA and R‐SAA patients are presented in Table 2. We performed correlation analyses between the mRNA levels of HATs in CD8+ T cells and the clinical parameters of peripheral blood and bone marrow in SAA and R‐SAA patients.

**TABLE 2:** Characteristics of SAA and R‐SAA patients

Characteristics	SAA (*N* = 20)	R‐SAA (*N* = 18)
Male/female	15/5	13/5
Age	40.5(15–79)	30(11–64)
Abnormal chromosome	Absence	Absence
PNH clone	None	None
Treatment	Untreated	ATG + CsA
WBC(*10^9^/L)	1.53 ± 0.17**	5.01 ± 0.56
Hb(g/L)	71.85 ± 2.59**	127.22 ± 8.13
PLT(*10^9^/L)	40.40 ± 6.13**	98.67 ± 16.76
Neu(%)	23.84 ± 3.89**	46.61 ± 3.72
ANC(*10^9^/L)	0.37 ± 0.07**	2.43 ± 0.42
Lym(%)	69.28 ± 4.42**	43.65 ± 3.84
Lym(*10^9^/L)	1.03 ± 0.13**	2.11 ± 0.23
Ret(%)	0.48 ± 0.08**	1.81 ± 0.23
Ret(*10^9^/L)	8.96 ± 1.53**	73.13 ± 9.05

Note

Age is expressed as median (range) and clinical parameters as mean ± SED.

Abbreviations: ANC—absolute neutrophil count; ATG—antithymocyte globulin; CsA—cyclosporin A; Hb—hemoglobin; Lym—lymphocyte; Neu—neutrophils; PLT—platelet; Ret—reticulocyte; SAA—severe aplastic anemia; R‐SAA—remission SAA patients; WBC—white blood cell.

**p < 0.01, compared with R‐SAA.

**FIGURE 3:**
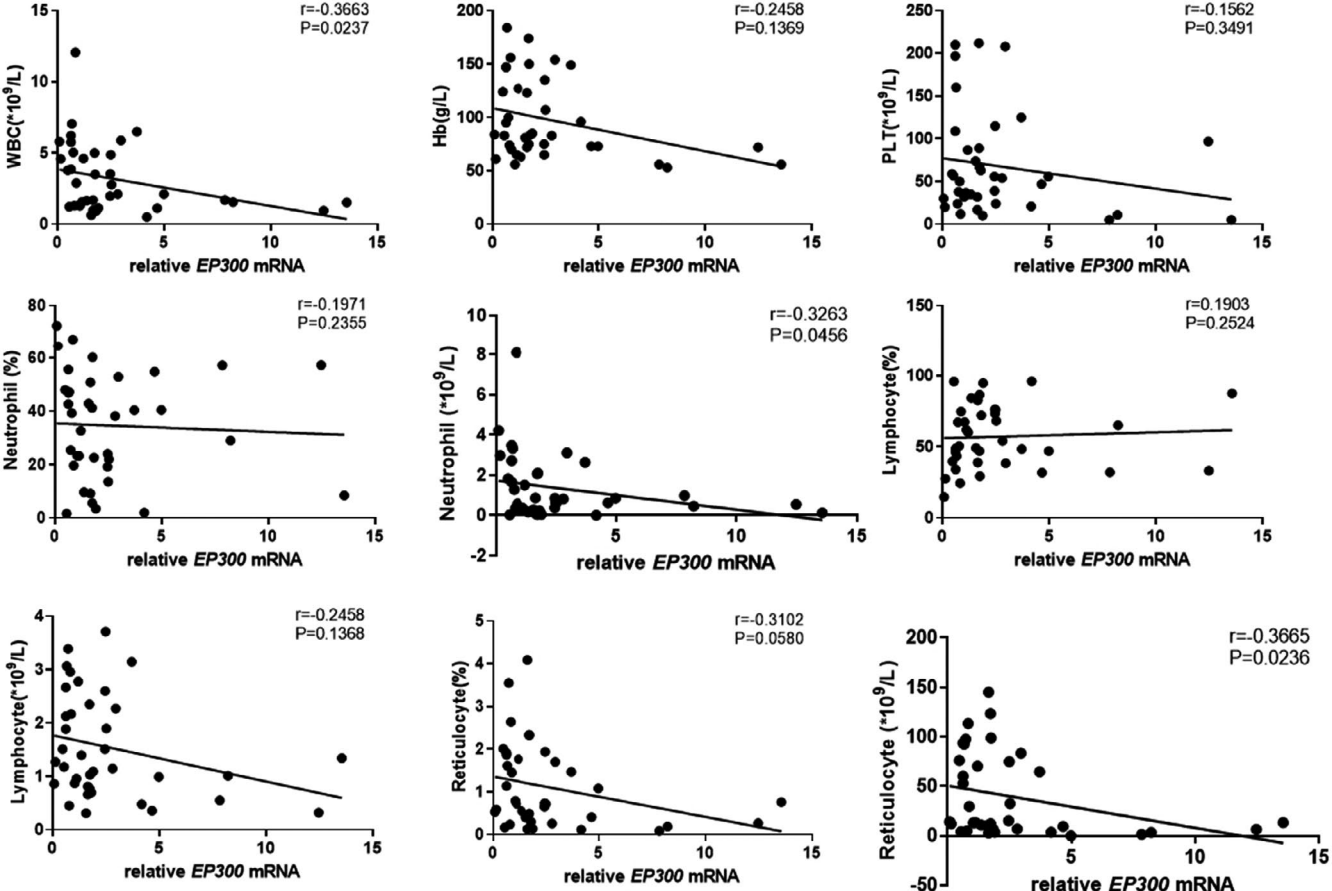
Correlation between *EP300* mRNA expression in peripheral blood CD8+ T cells and clinical parameters of peripheral blood in SAA and R‐SAA patients. SAA—severe aplastic anemia; R‐SAA—remission SAA patients

**FIGURE 4:**
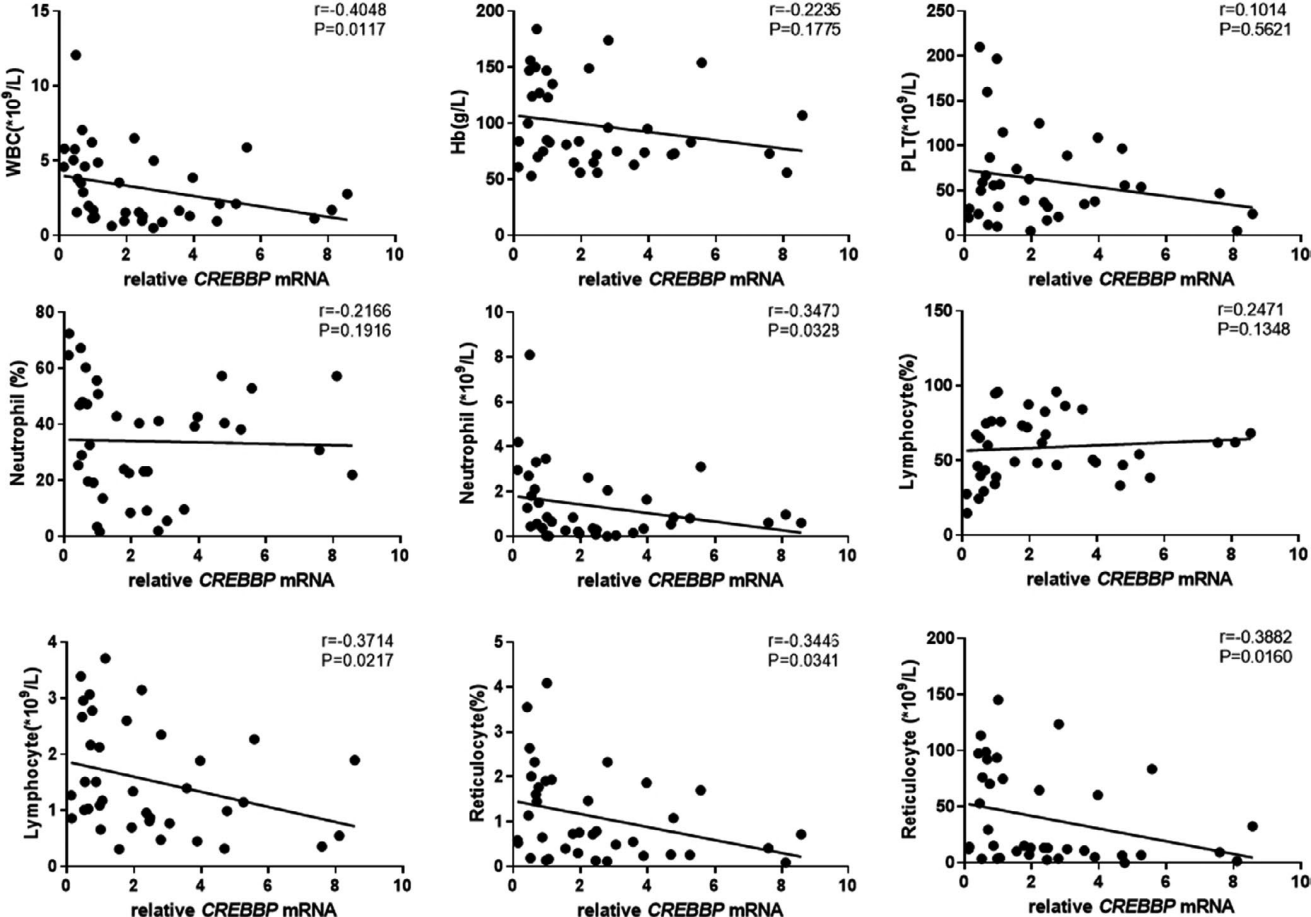
Correlation between *CREBBP* mRNA expression in peripheral blood CD8+ T cells and clinical parameters of peripheral blood in SAA and R‐SAA patients. SAA—severe aplastic anemia; R‐SAA—remission SAA patients

The results showed that *EP300* mRNA expression in peripheral blood CD8+ T cells of patients with SAA and R‐SAA was significantly and negatively correlated with the clinical parameters of peripheral blood, including the number of WBCs, ANC, and ARC (*p* < 0.05; Figure 3). *EP300* mRNA expression was also negatively correlated with hemoglobin, platelets, proportion of neutrophils, ALC, and proportion of reticulocytes, but without statistically significant differences. Similarly, *CREBBP* mRNA expression was negatively correlated with the number of WBCs, ANC, ALC, proportion of reticulocytes, and ARC in peripheral blood (*p* < 0.05; Figure 4). Furthermore, it was also negatively correlated with hemoglobin, platelets, and the proportion of neutrophils, but without statistically significant differences. To some degree, both of *EP300* and *CREBBP* mRNA expression were positively correlated with the percentage of lymphocytes, but those were not significant (Figures 3 and 4).

**FIGURE 5:**
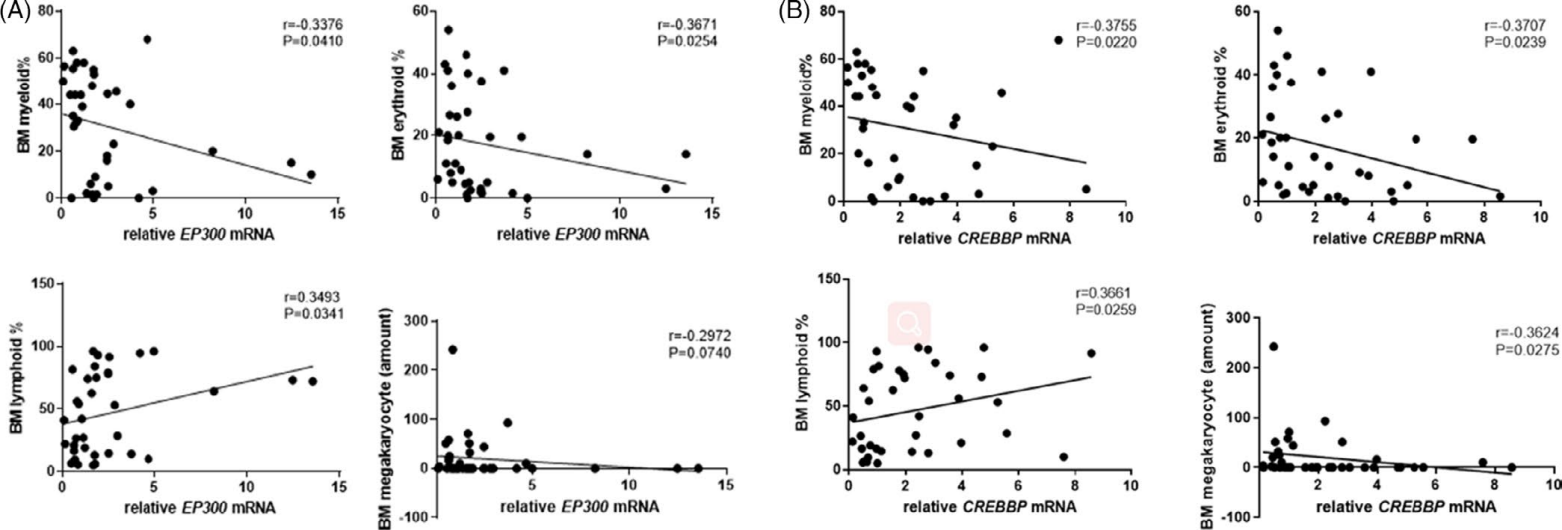
Correlation between (A) *EP300*, (B) *CREBBP* mRNA expression in peripheral blood CD8+ T cells, and clinical parameters of bone marrow in SAA and R‐SAA patients. SAA—severe aplastic anemia; R‐SAA—remission SAA patients

The mRNA expression of *EP300* in CD8+ T cells of patients with SAA and R‐SAA was significantly and negatively correlated with the percentage of bone marrow myeloid and erythroid cells, and it was significantly and positively correlated with the percentage of bone marrow lymphoid cells (*p* < 0.05; Figure 5A). *EP300* mRNA expression was also negatively correlated with the number of megakaryocytes, but this was not significant. Similarly, *CREBBP* mRNA expression in peripheral blood CD8+ T cells of patients with SAA and R‐SAA was significantly and negatively correlated with the percentage of bone marrow myeloid, erythroid cells, and the megakaryocyte count, and it was significantly and positively correlated with the percentage of bone marrow lymphoid cells (*p* < 0.05; Figure 5B).

## 4 DISCUSSION

Severe aplastic anemia is a hematological disease that is characterized by bone marrow failure and severe pancytopenia. The recognized pathogenesis of SAA is the destruction of hematopoietic cells by autoreactive T cells.^1^ In SAA patients, the number of CD8+ T cells are significantly increased and the ratio of CD4+ to CD8+ T cells is inverted. Activated CD8+ cytotoxic T lymphocytes were considered to be the most important effector T cells that inhibit hematopoiesis in SAA patients. CD8+ T cells exhibit enhanced functions by secreting type 1 lymphokines such as IFN‐γ, perforin, and granzymes, which could induce the apoptosis of hematopoietic cells and inhibit the hematopoiesis of bone marrow.^2^ However, the reason why CD8+ T cells can be activated continuously remains unclear.

In recent years, the result of epigenetic modifications that could regulate the activation of CD8+ T cells has implicated the role of epigenetic modifications of CD8+ T cells in some autoimmune diseases.^7^, ^8^ Therefore, we attempted to explore the immune pathogenesis of SAA from the perspective of epigenetic mechanisms. Our previous study^5^ revealed that the amount of histone H3 acetylation in CD8+ T cell of untreated SAA patients increased significantly, and it was significantly correlated with the disease severity. This result preliminarily indicated that abnormal histone H3 acetylation of CD8+ T cells may play a role in the immune pathogenesis of SAA.

Histone acetylation is a type of histone modification that involves covalent post‐translational modifications on residues located at histone tails. The covalent modifications of histones are catalyzed by specific histone‐modifying enzymes. Histone acetylation modifications are regulated by a balance between the activity of HATs and HDACs.^4^ HATs transfer acetyl groups to the amino groups of lysine residues from acetyl‐CoA, which induces a loosening of the chromatin structure and activates gene transcription. Conversely, HDACs catalyze hydrolysis of acetyl groups from the lysine residues, which leads to the formation of heterochromatin and transcriptional repression.

In this research, we investigated the mRNA expression of HATs and HDACs in CD8+T cells from SAA patients by RT‐PCR. The results suggested that mRNA of *EP300* and *CREBBP* was significantly increased in CD8+ T cells of SAA patients compared with the normal controls. Furthermore, there was a significantly positive correlation between the expression of *EP300* and *CREBBP* in CD8+ T cells of SAA. However, the expression of *HDAC1* and *HDAC7* mRNA had no significant differences between SAA patients and the healthy controls. Our research demonstrated the imbalance between HATs and HDACs in CD8+ T cells of SAA patients, which may be one of the reasons for the increase of histone H3 acetylation in SAA. In addition, the imbalance between HATs and HDACs was confirmed by another study on CD8+T cell transcriptomes. In that study, we detected the transcriptomics of peripheral blood CD8 + T cells from 4 SAA patients and 4 healthy controls. A total of 1274 genes were found to be significantly differentially expressed between SAA and healthy controls (fold change > 1.5, *p* < 0.05). *EP300* and *CREBBP* were differentially expressed genes, but there was no difference in the expression of *HDAC1* and *HDAC7*. The data of the study have not been published, and the heat map of differentially expressed genes was in the supplement file Figure S1.

Previous studies have proved that abnormal modification of histone acetylation, and abnormal histone‐modifying enzymes are involved in some autoimmune diseases. In systemic lupus erythematosus (SLE) patients, the acetylation of histone H3 and H4 was decreased in CD4+ T cells, and the level of histone H3 acetylation was significantly negatively correlated with the SLE severity.^9^ Meanwhile, *SIRT1* was overexpressed in the CD4+ T cells from active SLE patients, while the other HATs and HDACs were downregulated. In addition, the modification of histone H3 on the *CRE* site within the *IL17A* promoter was involved with H3K18 hyperacetylation and H3K27 hypomethylation in SLE T cells, which resulted from the recruitment of HDAC1 and DNA methyltransferase3a to the *CRE* site.^10^ In Graves' disease (GD), genome‐wide profiling of H3K27ac in CD8+ T cells of patients revealed that the acetylation level of H3K27 decreased in the promoter of inhibitory receptor genes, which may weaken the inhibitory effect of receptor signaling in CD8+ T cells.^11^ A study of histone modification in systemic sclerosis (SS) patients showed that global acetylation of histone H4 was upregulated in B cells, while HDAC2 and HDAC7 were significantly downregulated. Global histone H4 acetylation was negatively correlated with HDAC2 expression. Moreover, global H4 acetylation and HDAC2 protein expression were both correlated with SS activity.^12^

In the current study, we analyzed the correlation between the mRNA of *EP300* and *CREBBP* in CD8+ T cells and the disease severity of SAA patients. We retrospectively analyzed the complete blood counts and the percentage of hematopoietic cells in the bone marrow of SAA patients. In SAA and R‐SAA patients, a negative correlation was identified between *EP300* mRNA expression and clinical parameters of peripheral blood, including the levels of leukocytes, ANC, and ARC. Similar to *EP300*, *CREBBP* mRNA expression also showed a negative correlation with leukocytes, ANC, ALC, ARC, and proportion of reticulocytes. In addition, *EP300* and *CREBBP* mRNA expression was negatively correlated with bone marrow myeloid cells, erythroid cells, and megakaryocytes. Conversely, the expression was positively correlated with bone marrow lymphocytes. We concluded that the mRNA expression levels of *EP300* and *CREBBP* increased significantly at the onset of SAA, and the mRNA expression decreased gradually after immunosuppressive treatment. Therefore, the mRNA expression of *EP300* and *CREBBP* may be a potential indicator or biomarker of the disease severity and treatment response of SAA.

We also tested *IFNG* mRNA expression in CD8+ T cells from SAA patients and healthy controls. IFN‐γ plays an important role in the immune pathophysiology of hematopoietic failure in SAA. In aplastic anemia patients, intracellular IFN‐γ was overexpressed in circulating and bone marrow T cells. Furthermore, the IFN‐γ level change inside T cells may indicate the severity and relapse of aplastic anemia.^13^ In our study, the expression of *IFNG* mRNA in CD8+ T cells of untreated SAA patients increased significantly compared with the normal ones. Meanwhile, *EP300* and *CREBBP* mRNA expression showed a positive correlation with *IFNG* mRNA expression. Thus, we speculated that *EP300* and *CREBBP* may be involved in the overexpression of cytokines in CD8+ T cells. Our research was preliminary, and the related pathways and molecular mechanisms should be elucidated in the future studies.

In recent years, many researches have confirmed the important role of acetylation modifying enzymes in regulating T cell development, differentiation, activation, and function. Maekawa et al. found that p300 could bind to the promoter of granzyme B by forming a complex with Notch2 intracellular domain and transcription factor CREB1 in CD8+ T cells. Therefore, p300 may regulate the expression of granzyme B, which being important for the cytotoxicity of CD8+ T cells.^14^ Another study revealed that p300 interacting with signal transducer and activator of transcription 1 could increase the H3K27ac level in the enhancers of TNFAIP2 and LCP2, which may contribute to the development of inflammatory bowel disease, and the p300 inhibitor significantly inhibited the disease in an acute colitis mice model.^15^

Meanwhile, our study showed that the mRNA expression levels of *HDAC1* and *HDAC7* were also increased in SAA patients, but the differences were not significant. In previous study from our research team, the expression of SIRT1, a class III HDAC, was significantly decreased in CD8+ T cells of SAA, and it was significantly negatively correlated with IFN‐γ expression.^16^ On the basis of the above results, we speculated that HDAC1 and HDAC7 may not be the critical HDACs in SAA. The increased histone H3 acetylation in CD8+ T cells of SAA patients may be mainly caused by the increase in HATs and the decrease in class III HDACs.

In conclusion, elevated expression of histone acetylation enzymes p300 and CBP led to increased histone acetylation levels in CD8+ T cells of SAA patients, which contribute to the abnormal activation of CD8+ T cells. The imbalance between HATs and HDACs in CD8+ T cells may play a certain role in the immune pathogenesis of SAA. The above results are worthy of further exploration to clarify the pathogenesis of SAA from the perspective of epigenetic mechanisms, which are essential for developing targeted therapies for treatment of SAA patients.

## CONFLICT OF INTEREST

The authors declare that they have no conflict of interest.

## INFORMED CONSENTS

All patients or their relatives had to sign the informed consent before their data were recruited.

## Supporting information

Figure S1

## Data Availability

The data that support the findings of this study are available on request from the corresponding author.
