# Supplementary figures and images for: Abnormal expression of histone acetylases in CD8+ T cells of patients with severe aplastic anemia
Source: J Clin Lab Anal. 2022 Mar 10;36(4):e24339. doi: 10.1002/jcla.24339 (PMC8993608; doi:10.1002/jcla.24339)

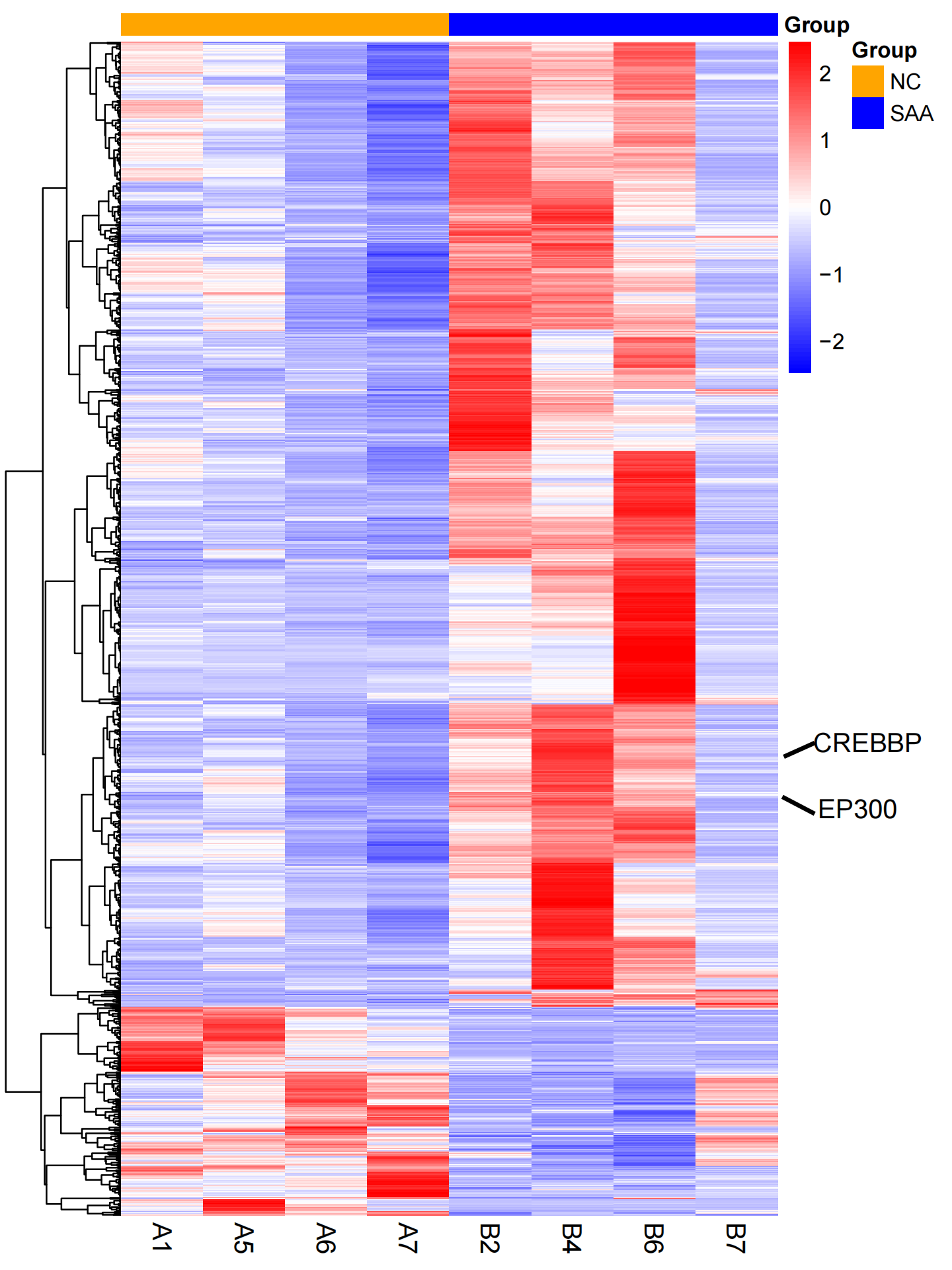

Supplement: Supplementary file 1 — Figure S1 [file JCLA-36-e24339-s001.tif]
